# Social network structure and composition in former NFL football players

**DOI:** 10.1038/s41598-020-80091-w

**Published:** 2021-02-01

**Authors:** Amar Dhand, Liam McCafferty, Rachel Grashow, Ian M. Corbin, Sarah Cohan, Alicia J. Whittington, Ann Connor, Aaron Baggish, Mark Weisskopf, Ross Zafonte, Alvaro Pascual-Leone, Albert-László Barabási

**Affiliations:** 1 Brigham and Women’s Hospital/Harvard Medical School, Boston, MA 02115 USA; 2 Department of Neurology, Brigham and Women’s Hospital, Boston, MA USA; 3 Network Science Institute, Northeastern University, Boston, MA USA; 4 Football Players Health Study at Harvard University, Boston, MA USA; 5 Department of Environmental Health, Harvard T.H. Chan School of Public Health, Boston, MA USA; 6 Department of Neurology, Berenson-Allen Center, Beth Israel Deaconess Medical Center, Boston, MA USA; 7 Department of Cardiology, Massachusetts General Hospital, Boston, MA USA; 8 Department of Physical Medicine and Rehabilitation, Spaulding Rehabilitation Hospital, Massachusetts General Hospital, Brigham and Women’s Hospital, Boston, MA USA; 9 Hinda and Arthur Marcus Institute for Aging Research and Center for Memory Health, Hebrew SeniorLife, Boston, MA USA; 10 Guttmann Brain Health Institut, Institut Guttmann, Universitat Autonoma Barcelona, Barcelona, Spain; 11 Department of Physics, Northeastern University, Boston, MA USA

**Keywords:** Brain injuries, Neurodegeneration, Outcomes research, Risk factors

## Abstract

Social networks have broad effects on health and quality of life. Biopsychosocial factors may also modify the effects of brain trauma on clinical and pathological outcomes. However, social network characterization is missing in studies of contact sports athletes. Here, we characterized the personal social networks of former National Football League players compared to non-football US males. In 303 former football players and 269 US males, we found that network structure (e.g., network size) did not differ, but network composition (e.g., proportion of family versus friends) did differ. Football players had more men than women, and more friends than family in their networks compared to US males. Black players had more racially diverse networks than White players and US males. These results are unexpected because brain trauma and chronic illnesses typically cause diminished social relationships. We anticipate our study will inform more multi-dimensional study of, and treatment options for, contact sports athletes. For example, the strong allegiances of former athletes may be harnessed in the form of social network interventions after brain trauma. Because preserving health of contact sports athletes is a major goal, the study of social networks is critical to the design of future research and treatment trials.

## Introduction

A personal social network is an individual’s family, friends, and acquaintances and their interpersonal connections. These networks, through various mechanisms ranging from social influence to neurohumoral cascades, have broad effects on health outcomes and quality of life^1^. In contact sports athletes, understanding the role of social networks could have significant treatment implications for mitigating brain trauma effects and improving quality of life. However, study of social networks has been limited and separate from longitudinal studies of this cohort^2^.

American-style football players are a particularly high-risk group who experience multiple chronic illnesses. Particularly, there have been concerns of neurodegenerative disorders such as chronic traumatic encephalopathy, Alzheimer’s disease, and amyotrophic lateral sclerosis^3,4^. These athletes also experience cardiovascular^5^, endocrine^6^, and mental health illnesses^7^ due to exposure to unique physiological stress and trauma. Examining sociality in the context of these illnesses is an unmet need.

Notably, not all football players experience these multiple medical disorders, despite similar brain trauma history. This observation suggests that the link between exposure and outcome is not clear-cut. Instead, the relationship is modified by other factors that elevate or reduce the risk for developing or manifesting disease. Biopsychosocial factors, including social network changes, retirement adjustments, and financial status, have been suggested as influential effect modifiers that require further study^2^.

For these reasons, we studied the personal social networks of former National Football League (NFL) players compared to non-football US males. Personal social network mapping, or egocentric network analysis, is a useful proxy for multiple social factors, including social support, access to resources, health habit influences, and time available to socialize^8,9^. Concretely, the mapping procedure identifies the specific persons in an individual’s social world one by one, their links to each other, and their demographic and health-related characteristics. In these analyses, network structure is defined as the quantitative description of the arrangement of social ties (e.g., network size and density). Network composition are metrics that summarize the characteristics of network members (e.g., percentage of kin and diversity of race). We have demonstrated the utility of this method in health outcomes research, including studies of stroke^10^ and multiple sclerosis^11^.

Our aim is to characterize the personal networks of former NFL players compared to non-football controls to provide a more contextualized view of this professional cohort. Given the exposure to brain trauma and burden of chronic illness in the athletes, we hypothesized that former NFL players would have constricted personal social networks compared to US controls. Contrary to this hypothesis, the findings show social network structure did not differ, but the types of persons who make up the players’ personal networks did differ from US controls.

## Results

### Health and demographic data of former NFL football players and US controls

We analyzed the results of 303 former football players and 269 controls (Table 1). Among the former football players, 129 (42.6%) reported a chronic health condition compared to 61 (22.7%) of controls. Other differences between the groups were that the former football players were older (59 [47–68] versus 38.5 [32–46]), more racially diverse (26.0% non-white versus 4.1%), and more likely to be retired (29.4% versus 0.8%). Both groups were highly educated compared to the general population, though football players had higher college education rates (92.6% versus 81.5%). Former football players were more likely to be married (82% versus 70%), but both groups tended to live with others (87% versus 86%). The median yearly income of both groups inferred from zip codes did not differ ($68 810 [53 052, 84 851] versus $65 884 [51 687, 83 622]).

**Table 1:** Baseline characteristics of participants.

Characteristic	Controls (n = 269)	Former football players (n = 303)	P-value
**Sex, no. (%)**			NA
Male	269 (100.0)	303 (100.0)	
Female	0 (0.0)	0 (0.0)	
**Age, (median [IQR])**	38.50 [32.00, 46.00]	59.00 [47.00, 68.00]	** < 0.001**
Race, no. (%)*			** < 0.001**
White	254 (95.8)	222 (73.3)	
Black	2 (0.8)	75 (24.8)	
Other	9 (3.4)	4 (1.3)	
**Education, no. (%)**			** < 0.001**
High school or less	6 (1.3)	0 (0.0)	
Some college	29 (10.9)	19 (6.3)	
Associate degree	14 (5.3)	3 (1.0)	
Bachelor's degree	115 (43.2)	184 (61.3)	
Graduate degree	102 (38.3)	94 (31.3)	
**Employment, no. (%)**			** < 0.001**
Employed	245 (92.5)	186 (62.2)	
Student	11 (4.2)	1 (0.3)	
Retired	2 (0.8)	88 (29.4)	
Unemployed	7 (2.6)	24 (8.0)	
**Median yearly income (median [IQR])**	65 884 [51 687, 83 622]	68 810 [53 052, 84 851]	0.424
**Domestic status, no. (%)**			***0.001***
Married	187 (69.9)	251 (82.8)	
Not married	81 (30.1)	52 (17.1)	
**Living situation, no. (%)**			0.895
Live with others	228 (86.0)	256 (86.8)	
Live alone	41 (14.0)	47 (13.2)	
**Chronic health condition, no. (%)**			** < 0.001**
Present	61 (22.7)	129 (42.6)	
Not present	182 (67.7)	174 (57.4)	

We compared the former football player respondents to non-respondents (Supplement Table 1). We found that non-responders were younger (52 [39.0–63.0] versus 56.0 [44.5–65.0]), and more likely to be non-white (42.3% versus 26%). There were no differences in marital status, employment, football experience, concussion symptoms, mood, weight, or smoking status.

### Social network structure and composition of former NFL football players and US controls

**Figure 1:**
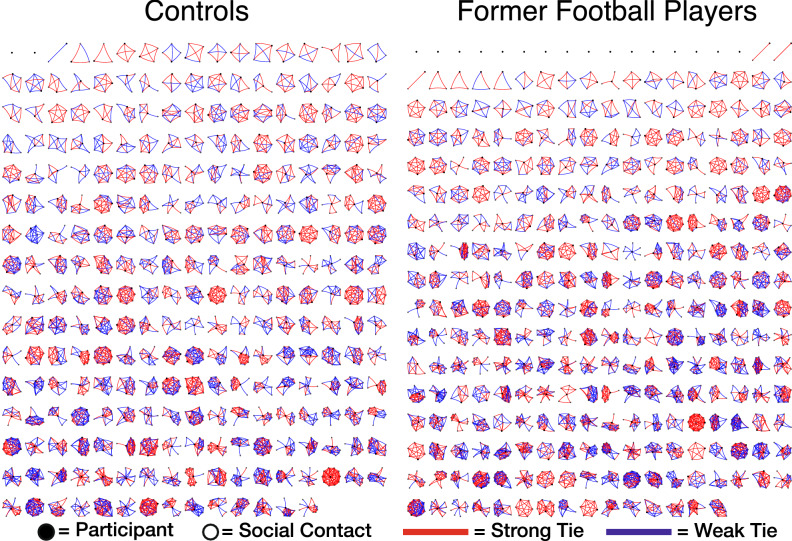
Personal networks of controls and former football players. Networks are arranged from smallest (top left) to largest (bottom right).

The former football players described 2575 network members, and the US controls described 2305 network members. A montage of all participants’ personal networks is displayed in Fig. 1, highlighting the range of small to large networks. Overall, the social networks were structurally similar across the two groups (Table 2, Supplement Fig. 1). Network size did not differ after accounting for age, race, education, employment status, median yearly income, and domestic status. The median, interquartile range [IQR] of network size for football players versus US controls was 8 [5, 11] versus 7 [6, 10], p = 0.499. All other metrics of network typology were likewise not statistically different.

**Table 2:** Network variables of former NFL football player and controls, adjusted.

	Controls (n = 269)	Former football players (n = 303)	P-value^c^
	Median [IQR]		
**Network structure variable**^**a**^			
Network size	7.00 [6.00, 10.00]	8.00 [5.00, 11.00]	0.499
Density	0.68 [0.50, 0.90]	0.75 [0.53, 0.96]	0.208
Constraint	47.22 [40.05, 57.73]	46.00 [36.68, 59.22]	0.239
Effective size	3.61 [2.43, 4.88]	3.50 [2.39, 5.07]	0.353
Max degree	5.00 [4.00, 7.00]	5.00 [4.00, 7.00]	0.456
Mean degree	3.71 [2.67, 5.00]	4.00 [2.67, 5.00]	0.282
**Network composition variable**^**b**^			
Percentage of kin	42.86 [33.33, 62.50]	40.00 [22.22, 60.00]	***0.045***
Diversity of sex	0.94 [0.83, 0.98]	0.84 [0.64, 0.96]	***0.001***
Diversity of race	0.00 [0.00, 0.26]	0.00 [0.00, 0.33]	0.825
Percentage of distant ties	33.33 [16.67, 50.00]	40.00 [20.00, 60.00]	0.121
Standard deviation of ages	12.91 [8.96, 15.96]	11.93 [7.62, 14.83]	0.791
Percentage of non-exercising ties	33.33 [14.29, 50.00]	30.00 [10.00, 50.00]	0.217
Percentage of negative ties	0.00 [0.00, 10.00]	0.00 [0.00, 0.00]	0.513
Percentage of persons who played organized football	–	40.00 [25.00, 60.00]	–
Percentage of persons who played in the NFL with participant	–	0.00 [0.00, 0.00]	–

^a^Network Structure is a quantitative description of the arrangement of social ties in each individual’s personal network. See definition of each term in “Methods”.

^b^Network composition is the range of characteristics of people around the individual. See definition of each term in “Methods”.

^c^P-value calculated from multivariable linear regression adjusting for age, race, education, employment status, median yearly income, and domestic status.

**Figure 2:**
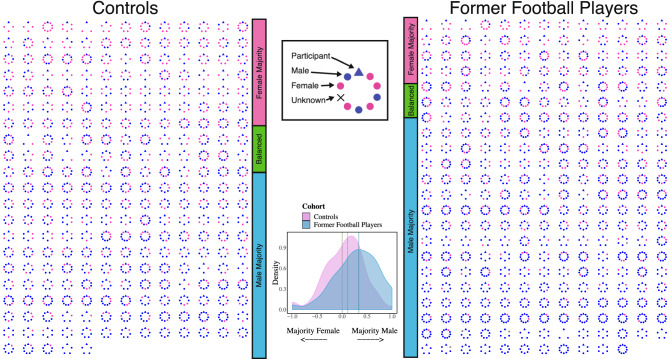
Male and female persons within networks of the two groups. Networks are ordered, from top left to bottom right, by the degree to which networks are majority female, balanced between female and male persons, or majority male. A summary of the sex distribution of the networks is visualized by a density plot. Participants with networks < 2 or entirely missing data are not included in the montages. Former football players are more likely to be surrounded by men than controls.

The composition of the networks differed across the groups in two ways (Table 2). Former football players had more men than women in their networks, as indicated by the lower median [IQR] Diversity of Sex (0.84 [0.64, 0.96] versus 0.94 [0.83, 0.98], p = 0.001) (Fig. 2). Secondly, the percentage of network members who were family members was less in the football players compared to controls (40.00% [22.22, 60.00] versus 42.86% [33.33, 62.50], p = 0.045). All other social network composition metrics were similar in the main comparison.

Football affiliations were only asked of the players cohort. Players’ networks had a median 40.00% [25.00, 60.00] of members who had played organized football (high school, college, professional). However, the networks rarely included members who played professional football. 196 participants (64.9%) had no NFL contacts, and 91 participants (30.0%) had only one NFL contact. In terms of playing with the participant, 14.29% [0.00, 33.33] of network members were former teammates or coaches in organized football. However, 0.00% [0.00, 0.00] were former teammates or coaches at the professional level.

### Network metrics stratified by race

We stratified the football players’ cohort into two groups, White (n = 222) and Black/Other (n = 79). We compared each group to each other, and then to controls, whose participants were 96% White. Network structure did not differ between the race sub-groups and controls, showing no difference from the full group findings (Supplement Table 2).

**Figure 3:**
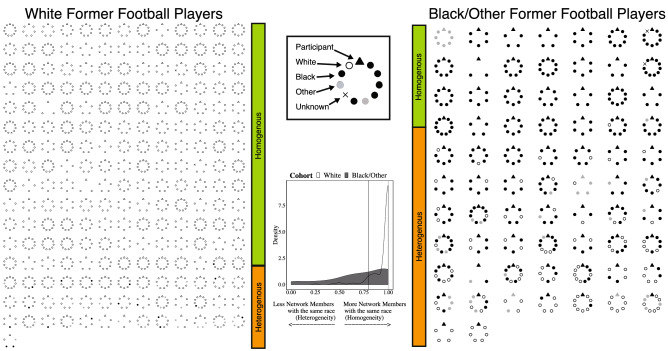
Racial makeup of networks in White versus Black/Other former football players. Networks are ordered by the degree to which networks have the same race as the respondent (top left, homogenous) to networks that have different race as the respondent (bottom right, heterogenous). A summary of the racial homogeneity versus heterogeneity distributions of the networks is visualized by the density graph. Participants with networks < 2 or entirely missing data are not included in the montages. Black/Other former football players are more likely than white former football players to have racially diverse networks.

There were differences in network composition in the race sub-groups, and particularly Diversity of Race (Fig. 3, Supplement Table 3). Black/Other former football players had a markedly higher diversity in their networks (0.42 [0.06, 0.78]) compared to White former football players (0.00 [0.00, 0.00]) and controls (0.00 [0.00, 0.26], p < 0.001). As displayed in Fig. 3, Black/Other football players were embedded in racially diverse (heterogenous) networks more than White football players who were embedded in racially uniform (homogenous) networks. This finding is distinct from the structural or other compositional patterns described below.

Other network composition findings are summarized in Supplement Table 3. Diversity of Sex did not differ across race sub-groups (White 0.83 [0.63, 1.03], Black/Other 0.89 [0.72, 1.06]), and but both were lower compared to the controls (0.94 [0.83, 0.98]). This suggested that race did not play a role in the tendency of football players to have more male social contacts. Percentage of Kin did not differ in White players (40.00 [22.22, 60.00]) compared to Black/Other (50.00 [22.92, 66.67]). However, White players, but not Black/Other players, had lower Percentage of Kin compared to controls (42.86 [33.33, 47.48]). Percentage of Distant Ties (persons more than15 miles from the participant) did not differ for White (44.00 [20.00, 60.00]) versus Black/Other (47.22 [28.57, 71.43]). However, Black/Other players had higher Percentage of Distant Ties compared to controls (33.33 [16.67, 50.00]).

### Network structure metrics stratified by number of and types of chronic illnesses

We performed a sensitivity analysis to test whether football players without health problems had more robust network structure than those with chronic illnesses. Such a pattern would suggest that the healthy subgroup elevated the network metrics of the cohort as whole. It would also suggest that football players began with more robust networks at baseline that diminish with chronic illnesses.

When stratifying those players without health problems (n = 174) versus those with one or more health problems (n = 129), there were no differences in network structure metrics (e.g., network size median [IQR] was 8 [5, 11] versus 7 [5, 10], p = 0.694) (Supplement Table 4). We additionally stratified for number of health problems as follows: no health problems (n = 179) versus one (n = 87), two (n = 30), and three or more (n = 12). There were no differences in the network metrics for each chronically ill group compared to the no health problem group (Supplement Table 5). Lastly, we stratified by types of health problems as follows: no health problem (n = 174), sleep apnea (n = 78), pain (n = 30), and cardiometabolic (n = 56). We found no significant differences in network structure metrics for each illness category against the no health problem group (Supplement Table 6).

In summary, sensitivity analysis suggested that football players without chronic illnesses have similar network structure as those with chronic illnesses.

## Discussion

Former NFL football players had social network structure that did not differ, and social network composition that did differ from US males. The football players’ networks had more men than women, and more friends than family in their networks compared to US males. Black/Other Race players had more racially diverse networks compared to White players and US males. The a priori hypothesis that players’ networks would be constricted in the context of brain trauma exposure and burden of chronic illness was not supported by these data.

These network structure findings differ from studies of social behavior in populations with brain trauma and other chronic illnesses. In a systematic review, an Institute of Medicine committee found that traumatic brain injury had clear adverse effects on social functioning in adults, particularly in terms of unemployment and diminished social relationships^12^. In the UK Household Longitudinal Study, Sacker et al. shows that poor health is related to subsequent social exclusion in a dose–response relationship^13^. In a study of older adults in a US metropolitan area, Finlay et al. report that physical and mental health resulted in higher reports of social isolation^14^. In a nationally represented US sample, Cornwell shows that poor health is related to more close-knit, constricted networks^15^. Finally, neurological diseases are particularly influential drivers. Patients with ischemic stroke have longitudinal declines in social networks^10,15^. Therefore, former football players appear to be outliers in terms of preserved sociality in the context of varying degrees of brain trauma and chronic illnesses.

The male-dominant, friendship-oriented, and racially diverse network pattern has been previously reported in studies of co-workers in workplaces^16,17^. Co-worker ties are more homogenous on sex and education and heterogeneous in race and religion. This is because the work environment provides opportunities to contact and establish relations among non-family associates^18^. In the case of former football players, this pattern may reflect fraternal bonds structured by football, but not composed of prior professional football contacts. Only a minority of networks members were alumni connections to professional teammates or coaches. Such homogenous ties have also been shown to be more resistant to loss over time^19^. This network mechanism may be one reason for the preserved network structure seen in our results. However, the persistence and resiliency of such workplace-like networks many years after retirement from professional sports are intriguing areas for further study.

The results have implications for understanding and interpreting later life neurological outcomes of former football players. Large social networks are associated with longevity^20^ and reduced incidence of dementia in a variety of populations^21^. There are multiple mechanisms, but one is buffering of stress and biological changes^22^. For example, social networks modify the relation between Alzheimer’s disease pathology and level of cognitive function^23^. Former football players may be benefiting from protective social network effects that should be considered in epidemiological and clinical-pathological studies.

These results also have clinical implications. First, the causal model of repetitive brain trauma leading to clinical and neuropathological outcomes needs revision. Biopsychosocial factors, including social relationships, likely alter the recovery and aging of the brain after trauma. This leads to varied phenotypic presentations. Appreciating biopsychosocial influences is critical when interpreting past results and designing future trials^2^. Second, unlike trauma, clinicians have a chance to modify social relationships. This may take the form of identifying persons who are socially isolated after brain trauma, and offering them social support, and/or treatment of associated neuropsychiatric symptoms via pharmacotherapy or psychotherapy^24^. Finally, an unexplored area is treatment development in the form of novel social network interventions after brain trauma. Using the strong allegiances of former athletes, social connectedness therapies harnessing social skills, social integration, and social interaction tracking may be developed. Researchers in addiction have shown early signals of efficacy of such approaches in similar populations^25^.

The strengths of our study include the use of personal network analysis to quantify the social contexts of professional football players versus US controls using the same instrument. We also carefully controlled for known demographic, socioeconomic, and clinical factors that may influence network dimensions. Finally, we completed various sensitivity analyses that strengthened our findings.

Our study has limitations. First, the two groups were not matched by sociodemographic factors, such as age and race. This was because we wanted to compare groups who filled out the same social network survey. Although we controlled for these covariates, the effects of these factors and unknown confounders may still be present. Notably, many of the differing characteristics, such as age and retirement status, should lead to smaller networks in the players^26^. Moreover, some of the effect sizes in the results, such as the difference in percentage kin, may be influenced by multiple comparisons. Second, participants who were more sociable or cognitively higher function may have completed the survey. Our analysis of responders versus non-responders suggested that the responders were older and Whiter than the Football Players Health Study at Harvard University cohort as a whole. This limits generalizability of the results. Also, the survey relied on patients’ self-reports of their social networks. Although participants’ report of their personal networks of intimate contacts have been shown to be accurate^27^, recall bias may still would limit accuracy. We do not see this limitation affecting one group preferentially. Lastly, as a cross-sectional study, network change could not be assessed.

In summary, former NFL players had personal social networks that were compositionally distinct but not structurally different from US males. These results are unexpected because brain trauma and chronic illnesses typically cause diminished social relationships. Moreover, there were unique features to the players’ networks: they had higher proportions of men and friends, and Black/Other Race players had more racially diverse networks. These prognostically positive features may modify the risk of developing or manifesting neurological disease, and therefore need to be included in longitudinal studies. Treatments that incorporate these robust social resources may also be promising.

## Methods

### Study design and participants

This study was a cross-sectional investigation of two cohorts who completed the same social network survey. The first was a group of former NFL football players currently enrolled in the Football Players Health Study at Harvard University (FPHS) who completed the survey in 2019. The second was a group of non-football US controls enrolled in the Genes and Environment in Multiple Sclerosis study who completed the survey in 2017^11^.

The FPHS cohort were former football players who had participated in the NFL after 1960. All participants played in the league after the adoption of hard-shelled helmets. As of April 2019, the cohort consisted of 3506 former football players who participated in a prior survey called the Health and Wellness Survey^28^. Of this group, 336 responded to our invitation to complete the Personal Network Survey. We removed 33 records due to incomplete or verified inaccurate survey responses, resulting in a final tally of 303 participants.

The US controls were a cohort of asymptomatic male family members of patients with Multiple Sclerosis from a prior study^11^. Because the study was related to the risk of multiple sclerosis, a disease more prominent in women, there were more women than men. Of the original 1493, the final tally of male controls was 269 individuals.

All participants provided informed consent. The Beth Israel Deaconess Medical Center institutional review board approved the project as part of the FPHS. The Partners HealthCare institutional review board approved the Genes and Environment in Multiple Sclerosis study. All research was performed in accordance with relevant guidelines/regulations.

### Network instrument

The Personal Network Survey for Clinical Research (PERSNET) is a scalable online tool on the REDCap platform^11^. The instrument, adapted from the General Social Survey^29^, contains three sections: name generator, network inter-relater, and name interpreter. The name generator asks three questions paraphrased as follows: “Who do you typically discuss important matters with?”, “Who do you often socialize with?” and “Who provides support for your health needs?” The participant lists an unlimited number of names, allowing network size to be calculated without a ceiling. In the network inter-relater section, the participant is asked to describe the presence and strength of inter-connections of the first 10 network members. In the name interpreter section, the participant is asked to describe all network members’ demographics, health habits, and affiliations with the participant. Please see Supplement Methods 1 for instrument.

### Data collection

We collected data using an electronically delivered survey on the REDCap platform for both cohorts. Participants received an email with a secure ID-locked link that provided access to the instrument. Participants completed the survey over ~ 10–20 min on a computer or mobile device. For the football players cohort, upon completion, participants received an email within 24 h that provided detailed information on their own personal network. It included a network map, summaries of select network characteristics, and information from experts on the general effects of networks on health (Supplement Methods 2). We employed a quality control procedure to identify cases of incomplete, inaccurate, or non-existent network data. For flagged records, our staff contacted participants and provided instructions to complete the survey correctly.

For additional demographic, football exposures, and health data, we used the Health and Wellness survey completed by participants between 2015 and 2019^28^. Data included race, marital status, football experience, concussion symptoms, mood, current body mass index, and smoking status. For chronic health conditions, we used the definitions of medical afflictions from a prior study of this cohort^30^. Neurocognitive affliction was having been diagnosed with dementia or chronic traumatic encephalopathy, or having ever been prescribed medication for memory loss. Pain affliction was having been prescribed pain medication for chronic pain being taken at the time of survey. Cardiometabolic affliction was having been diagnosed with a heart attack or stroke, or taking medication at time of survey for at least two of the following: diabetes, hypertension, or high cholesterol. Sleep affliction was having been diagnosed sleep apnea. For the US controls, we assigned them as having a chronic health condition if they stated yes to any symptoms that chronically affected walking, using arms and hands, vision, speech, swallowing, thinking, memory or concentration, numbness, tingling, burning sensation or pain, controlling bladder or bowel^31^.

### Network analysis

We analyzed two main categories of personal network metrics: network structure, which is the quantitative description of the arrangement of social ties; and network composition, which include metrics that summarize the characteristics of network members.

Within network structure, *Network Size* is the number of individuals in the network, excluding the participant. *Density* is the number of actual connections among individuals in the network divided by the number of possible connections in a network, excluding the survey participant. Similar to *Density*, *Constraint* is the degree to which each network member is connected to the others in the network, with additional benefits of incorporating hierarchies and strength of ties. *Effective Size* is the number of nonredundant members in the network, conceptually an inverse metric of *Constraint*. *Maximum Degree* is the highest number of ties by a network member, excluding the participant. *Mean Degree* is the average number of ties of a network member, indicating the distribution of ties in the network. Equations to calculate these measures are provided in Supplement Methods 3^32^.

Within network composition, we focused on key features that define the social and health milieu surrounding the participants and available in both datasets. *Diversity of Sex* is the mix of men and women in the network, based on the index of qualitative variation^33^. A value of 0 means all network members are one sex and a value of 1 means there is an equal number of men and women in the network. *Diversity of Race* is similarly the mix of races in the network, with a value of 0 indicating all persons are the same race and 1 indicating an equal proportion of each racial category (“White”, “Black’, and “Other”). *Percentage of Distant Ties* is the percentage of people who live more than 15 miles from the participant. *Percentage of Kin* is the percentage of people who are family (including spouse). *Standard Deviation of Ages* is the range of ages of network members. *Percentage of Non-Exercising Ties* is the percentage of people who do not exercise at least 3–4 times per week. *Percentage of Negative Ties* is the percentage of network members who the participant reported as having a negative influence on his health. *Percentage of Persons who Played Organized Football* or *Percentage of Persons who Played in the NFL with the Participant* are the percentage of network members who meet these characteristics.

We further investigated whether or not participants were surrounded by individuals who were like them, a feature known as homogeneity^16^. *Majority Male Score* was the number of men minus the number of women divided by the total number of network members, ranging from − 1 (Majority Female) to 1 (Majority Male). *Proportion of Network Members with the Same Race as the Participant* ranges from 0 (All different race, heterogenous) to 1 (All same race, homogenous).

### Statistical analysis

We compared the median values and categorical distributions between the two cohorts. First, we used univariate approaches, Mann–Whitney and Chi-Squared Tests, to assess for significant differences (measured as p < 0.05) in continuous and categorical data, respectively. Next, we used multivariate linear regression to compare the groups after adjusting for the covariates. In this regression, the dependent variable was the network metric (e.g., network size) and the independent variables were the group (e.g., football player or US control) and covariates. The covariates included age, race, education, employment status, median yearly income, and domestic status. These covariates were either significantly different between the two groups in univariate analysis (Table 1), or they were described by prior literature to be influential on social network formation^34^. Finally, we stratified according to race and chronic illness number and types. All analyses were completed in R version 3.6.1^35^.

## Supplementary Information

Supplementary Information

## Data Availability

Underlying data for the former NFL football players are confidential. Data for the US controls can be made available upon request. STROBE checklist attached (Supplement Methods 4).

## Code availability

The instrument, “Personal Network Survey for Clinical Research,” is available in the REDCap Shared Library. We have also uploaded a comprehensive R codebase for researchers who use the instrument to analyze and visualize their data available at: https://github.com/AmarDhand/PersonalNetworks. The R code, customized from the above codebase for this project, can be made available upon request.
