# Supplementary material for: Social network structure and composition in former NFL football players
Source: Sci Rep. 2021 Feb 1;11:1630. doi: 10.1038/s41598-020-80091-w (PMC7851122; doi:10.1038/s41598-020-80091-w)
Supplement: Supplementary file 1 — Supplementary Information [file 41598_2020_80091_MOESM1_ESM.pdf]

Social Network Structure and Composition in Former NFL Football Players

Amar Dhand, Liam McCafferty, Rachel Grashow, Ian M. Corbin, Sarah Cohan, Alicia J. Whittington, Ann Connor, Aaron Baggish, Mark Weisskopf, Ross Zafonte, Alvaro Pascual-Leone, Albert-László Barabási

Supplementary Table 1. Baseline Characteristics of Former Football Players Responders versus Non-Responders

Characteristic	Responders (n = 303)	Non-Responders (n = 3778)	P-value
Sex, No. (%)			
Male	303 (100.0)	4081 (100.0)	1.00
Female	0 (0.0)	0 (0.0)	
Age, (median [IQR])	56.0 [44.5-65.0]	52 [39.0-63.0]	**<0.001**
Race, No. (%)*			**<0.001**
White	222 (73.3)	2130 (56.4)	
Black	75 (24.7)	1480 (39.2)	
Other	4 (1.3)	119 (3.1)	
Missing	2 (0.7)	49 (1.3)	
Marital Status, No. (%)			
Married	236 (77.9)	2862 (75.7)	0.66
Not Married	64 (21.1)	883 (23.4)	
Missing	3 (1.0)	33 (0.9)	
Employment, No. (%)			
Unemployed	40 (13.2)	507 (13.4)	0.68
Yes, in football	27 (8.9)	371 (9.8)	
Yes, outside of football	184 (60.7)	2169 (57.4)	
Retired	50 (16.5)	677 (17.9)	
Missing	2 (0.7)	54 (1.5)	
Seasons of play (median [IQR])	5.0 [3.0-9.0]	6.0 [4.0-9.0]	0.10
Position			
Non-lineman	188 (62.0)	2472 (65.4)	0.23
Lineman	115 (38.0)	1306 (34.6)	
Concussion symptoms			

None	15 (5.0)	181 (4.8)	0.90
One or more	288 (95.0)	3596 (95.2)	
Mood category			
None	235 (77.6)	2919 (77.3)	
Depressed only	8 (2.6)	110 (2.9)	0.97
Anxious only	12 (4.0)	168 (4.4)	
Both depressed and anxious	47 (15.5)	574 (15.2)	
Missing	1 (0.3)	6 (0.2)	
Current Body Mass Index			
<25.0	23 (7.6)	194 (5.1)	0.25
25.0-30.0	122 (40.3)	1988 (52.6)	
>30.0	157 (51.8)	1566 (41.5)	
Missing	1 (0.3)	30 (0.8)	
Smoking status			
Never	251 (82.9)	3138 (83.0)	0.51
Former	7 (2.3)	120 (3.2)	
Current	44 (14.5)	486 (12.9)	
Missing	1 (0.3)	34 (0.9)	

Supplementary Figure 1: Density plots of select network structure variables across two groups

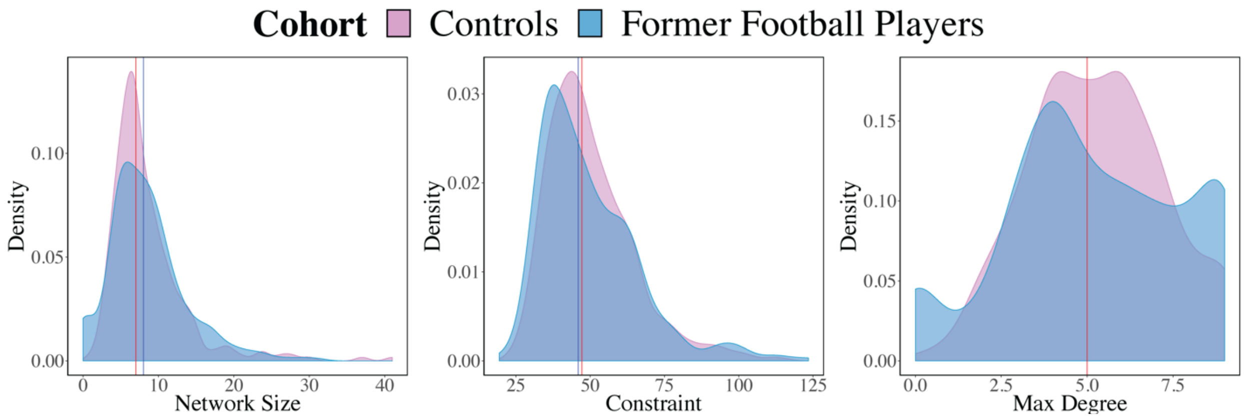

The plot represents the distribution of each network variable's values, colored by cohort. Network structure is similar across the two groups.

Supplementary Table 2: Network Structure Variables of Former NFL Football Player Stratified by Race and Compared to Controls, Adjusted

	Controls (n = 269)	Former Football Players (n = 301)			
		White Cohort (n = 222)		Black/Other Cohort (n = 79)	
	Median [IQR]	Median [IQR]	P-value[a]	Median [IQR]	P-value[a]
Network Size	7.00 [5.00, 9.00]	8.00 [5.00, 11.00]	0.605	7.00 [5.00, 9.00]	0.215
Density	0.68 [0.48, 0.88]	0.73 [0.53, 0.93]	0.092	0.80 [0.55, 1.05]	0.717
Constraint	47.22 [47.22, 56.06]	45.49 [34.67, 56.32]	0.392	46.42 [34.61, 58.23]	0.386
Effective Size	3.61 [2.38, 4.83]	3.69 [2.32, 5.07]	0.576	3.15 [1.96, 4.33]	0.630
Max Degree	5.00 [3.50, 6.50]	5.00 [3.00, 7.00]	0.395	4.00 [2.25, 5.75]	0.758
Mean Degree	3.71 [2.55, 4.88]	4.00 [2.83, 5.17]	0.188	3.25 [2.04, 4.46]	0.869

[a] P-value comparing each cohort of former football players to controls. It is calculated from multivariable linear regression adjusting for age, race, education, employment status, median yearly income, and domestic status.

Supplementary Table 3: Network Composition Variables of Former NFL Football Player Stratified by Race and Compared to Controls, Adjusted

	Controls (n = 269)	Former Football Players (n = 297)			
		White Cohort (n = 222)		Black/Other Cohort (n = 79)	
	Median [IQR]	Median [IQR]	P-value[a]	Median [IQR]	P-value[a]
Diversity of Sex	0.94 [0.83, 0.98]	0.83 [0.63, 1.03]	***0.002***	0.89 [0.72, 1.06]	***0.015***
Percentage of Kin	42.86 [33.33, 62.50]	40.00 [22.22, 60.00]	***0.041***	50.00 [22.92, 66.67]	0.564
Diversity of Race	0.00 [0.00, 0.26]	0.00 [0.00, 0.00]	0.988	0.42 [0.06, 0.78]	***<0.001***
Percentage of Distant Ties	33.33 [16.67, 50.00]	40.00 [20.00, 60.00]	0.164	47.22 [28.57, 71.43]	***0.015***
Average Age Deviation	12.91 [8.96, 15.96]	12.29 [8.56, 16.02]	0.913	10.61 [7.26, 13.95]	0.164
Percentage of Non-Exercising Ties	33.33 [14.29, 50.00]	33.33 [11.80, 50.00]	0.230	25.00 [0.00, 50.00]	0.674
Percentage of Negative Ties	0.00 [0.00, 10.00]	0.00 [0.00, 0.00]	0.352	0.00 [0.00, 0.00]	0.410

[a] P-value comparing each cohort of former football players to controls. It is calculated from multivariable linear regression adjusting for age, race, education, employment status, median yearly income, and domestic status.

Supplementary Table 4: Comparison of Network Structure Variables between Former NFL Football Players with Health Afflictions and No Health Afflictions, Adjusted

Network Structure Variable[a]	No Health Afflictions (n = 174)	Health Afflictions (n = 129)	P-value[b]
	Median [IQR]		
Network Size	8.00 [5.00, 11.00]	7.00 [5.00, 10.00]	0.694
Constraint	44.81 [36.23, 58.79]	46.19 [38.02, 59.23]	0.905
Density	0.73 [0.51, 1.00]	0.76 [0.53, 0.94]	0.705
Effective Size	3.56 [2.40, 5.21]	3.48 [2.35, 5.01]	0.773
Max Degree	5.00 [4.00, 7.00]	5.00 [4.00, 7.00]	0.916
Mean Degree	4.00 [2.80, 5.00]	4.00 [2.57, 5.00]	0.693

[a] Network Structure is a quantitative description of the arrangement of social ties in each individual's personal network. See definition of each term in Methods.
[b] P-value calculated from multivariable linear regression adjusting for age, race, education, employment status, median yearly income, and domestic status

Supplementary Table 5: Comparison of Network Structure Variables between Former NFL Football Players with No Health Afflictions and Former NFL Football Players with Increasing Number of Health Afflictions

Network Structure Variable[a]	No Health Afflictions (n = 174)		1 Health Afflictions (n = 87)		2 Health Afflictions (n = 30)		3+ Health Afflictions (n = 12)	
	Median [IQR]	P-value[a]	Median [IQR]	P-value[a]	Median [IQR]	P-value[a]	Median [IQR]	P-value[a]
Network Size	8.00 [5.00, 11.00]	*ref*	7.00 [5.00, 10.00]	0.675	7.50 [5.00, 10.00]	0.688	4.50 [1.50, 7.50]	0.311
Constraint	44.81 [36.23, 58.79]	*ref*	46.84 [38.17, 58.26]	0.795	42.80 [37.98, 58.60]	0.947	45.70 [38.05, 94.67]	0.066
Density	0.73 [0.51, 1.00]	*ref*	0.75 [0.58, 0.92]	0.746	0.75 [0.47, 0.89]	0.612	0.95 [0.58, 1.00]	0.451
Effective Size	3.56 [2.40, 5.21]	*ref*	3.50 [2.40, 5.00]	0.726	3.44 [2.13, 4.90]	0.912	3.29 [1.20, 5.21]	0.595
Max Degree	5.00 [4.00, 7.00]	*ref*	5.00 [4.00, 7.00]	0.761	5.00 [4.00, 7.00]	0.950	1.50 [0.75, 6.50]	0.253
Mean Degree	4.00 [2.80, 5.00]	*ref*	4.00 [2.67, 5.13]	0.644	4.00 [2.76, 4.96]	0.910	1.50 [0.21, 4.62]	0.403

[a] Network Structure is a quantitative description of the arrangement of social ties in each individual's personal network. See definition of each term in Methods.
[b] P-value calculated from multivariable linear regression adjusting for age, race, education, employment status, median yearly income, and domestic status

Supplementary Table 6: Comparison of Network Structure Variables between Former NFL Football Players with No Health Afflictions and Former NFL Football Players with Specific Health Afflictions

	No Health Afflictions (n = 174)		Sleep Apnea Affliction (n = 78)		Pain Affliction (n = 30)		Cardio-Metabolic Affliction (n = 56)		Neuro-Cognitive Affliction (n = 20)	
Network Structure Variable[a]	Median [IQR]	P-value[a]	Median [IQR]	P-value[a]	Median [IQR]	P-value[a]	Median [IQR]	P-value[a]	Median [IQR]	P-value[a]
Network Size	8.00 [5.00, 11.00]	*ref*	7.00 [5.00, 10.00]	0.722	7.00 [5.00, 9.75]	0.446	7.00 [5.00, 10.25]	0.565	5.00 [1.50, 7.50]	0.215
Constraint	44.81 [36.23, 58.79]	*ref*	45.87 [38.05, 57.13]	0.897	46.12 [40.04, 64.25]	0.052	46.55 [37.45, 62.97]	0.728	43.66 [38.25, 60.63]	0.909
Density	0.73 [0.51, 1.00]	*ref*	0.76 [0.53, 0.91]	0.803	0.83 [0.61, 1.00]	0.223	0.70 [0.53, 1.00]	0.802	0.80 [0.54, 1.00]	0.887
Effective Size	3.56 [2.40, 5.21]	*ref*	3.38 [2.38, 5.05]	0.848	3.29 [1.80, 4.78]	0.169	3.60 [1.96, 5.15]	0.922	3.29 [2.29, 4.32]	0.659
Max Degree	5.00 [4.00, 7.00]	*ref*	5.00 [4.00, 7.00]	0.866	4.00 [3.00, 6.75]	0.729	5.00 [3.00, 8.00]	0.789	3.00 [0.00, 6.00]	0.148
Mean Degree	4.00 [2.80, 5]	*ref*	4.00 [2.60, 4.96]	0.907	3.89 [2.50, 5.15]	0.985	3.90 [2.48, 5.06]	0.871	2.40 [0.00, 4.17]	0.421

[a] Network Structure is a quantitative description of the arrangement of social ties in each individual's personal network. See definition of each term in Methods.
[b] P-value calculated from multivariable linear regression adjusting for age, race, education, employment status, median yearly income, and domestic status

Supplementary Methods 1: Network Instrument

Persnet Survey

study_id ______________________________

We have included here for your review the Football Players Health Study Personal Network Study Informed Consent Sheet.

This document contains more information about the study and may help to answer any other questions you might have before completing the survey.

Please review this document before taking part in the study.

How do I agree to participate?

After reviewing the Informed Consent document, if you continue with this survey and submit your answers, you are saying that:

You agree to take part in this researchYou feel like you understand what you are getting intoYou understand that you are free to leave the study at any time

Tell us a little about yourself!

Your Age ______________________________

Race
(Select all that apply)

- ☐ Black or African American
- ☐ White
- ☐ American Indian/Alaska Native
- ☐ Asian
- ☐ Native Hawaiian or Other Pacific Islander
- ☐ Other
- ☐ Skip question

Ethnicity

- ◯ Hispanic or Latino
- ◯ NOT Hispanic or Latino
- ◯ Unknown
- ◯ Skip question

Education level

- ◯ Some high school or less
- ◯ High school grad
- ◯ Some college
- ◯ Associate degree
- ◯ Bachelor's degree
- ◯ Graduate degree
- ◯ Prefer not to answer

Your Zip Code ______________________________

	Yes - I work in Football	Yes - I work outside of Football	Retired	No	Student	Looking for work

Are you currently employed? (Select all that apply)	☐ ☐ ☐ ☐ ☐ ☐
Current Occupation	◯ Executive, manager<br>◯ Sales or clerical worker<br>◯ Mechanic, electrician, skilled worker<br>◯ Machine operator, inspector, bus/cab driver<br>◯ Service worker (e.g., janitor, guard)<br>◯ Professional (e.g., nurse, lawyer, teacher, etc.)<br>◯ Business owner<br>◯ Laborer, unskilled worker<br>◯ Farming<br>◯ Military<br>◯ Other
What is your approximate household net worth?	◯ less than $5,000<br>◯ $5,000 to $49,000<br>◯ $50,000 to $169,000<br>◯ $170,000 to $499,000<br>◯ more than $500,000<br>(This is the value of all the assets of people in your household (like housing, cars, stock, retirement funds, and business ownership) minus any debt or loans you and household members may have (like mortgages, credit card debt or car, school, or business loans). This does not include your regular income.)
How many people are in your household?	__________________________

Who is in your Personal Network?

In order to understand your personal network and the different ways in which people are important to you and support you, we are now going to help you create a master list of people by asking you who falls into these three categories:

Who do you discuss important personal matters with?Who do you often socialize with?Who provides you support for your health needs?
We are only interested in people who fall into one of those three categories, and we'd like you to think only about your current network of people in your life who are age 18 or older.

Please only use first names, nicknames, or initials - DO NOT USE FULL NAMES. If two people have the same first name, then add a last initial. You can enter any identifier you'd like in place of a name - all that matters is that you know the person you mean, as the "names" you enter will be used later in the survey. Even though they might not be full or real names, this information will be kept strictly confidential between you and the Harvard Football Players Health Study.

- - - - - - - - - - - -

From time to time, most people discuss important personal matters with other people. Who do you typically do that with?

Please do not list full names.

Name 1 ______________________

Name 2 ______________________

Name 3 ______________________

Name 4 ______________________

Name 5 ______________________

More names, separated by commas (if needed): ______________________

Who do you often socialize with? For example, these are people with whom you go out to dinner, go on a trip, or hang out.

Please do not list full names, and don't worry about duplicates from the previous section.

Name 1 ______________________

Name 2 ______________________

Name 3 ______________________

Name 4 ______________________

Name 5 ______________________

More names, separated by commas (if needed): ______________________

Think about people who encourage you to stay healthy by giving you motivation, advice, or direct help. Who provides this kind of support for your health?

Please do not list full names, and don't worry about duplicates from the previous section.

Name 1 ______________________

Name 2 ______________________

Name 3 ______________________

Name 4 ______________________

Name 5 ______________________

More names, separated by commas (if needed): ______________________

Finalizing your list of people:

Now, we are going to create a master list from the names you provided above. Know that we are only going to use the first 5 names you entered from each section above.

Please read these instructions carefully:For every individual person listed, click the "Keep" button next to the first time their name appears in the list.
If a person is listed more than once, only choose "Keep" once.Make sure you click "Keep" for each unique identity. If you do not click "Keep," then the name will be removed.All blanks and duplicates should be left as "Remove".

	Keep	Remove
[name1]	○	○
[name2]	○	○
[name3]	○	○
[name4]	○	○
[name5]	○	○
[name6]	○	○
[name7]	○	○
[name8]	○	○
[name9]	○	○
[name10]	○	○

[name11]	◯	◯
[name12]	◯	◯
[name13]	◯	◯
[name14]	◯	◯
[name15]	◯	◯

Number Filled in First 10 Slots

____
(This is just for us to keep track.)

Total number of social contacts

____
(Total number of names you kept.)

Relationship Description

We are now going to focus on the relationships in your personal network. If you notice some names missing, that is okay - we are only using the first 10 from your master list.

Below should be a list of unique names without blanks. If there are missing names from the first 10, duplicate names, or blanks, please STOP and go back to the previous section where you made your master list by choosing "Keep" for certain names. Please review the instructions for that section, and adjust your "Keep" or "Remove" choices as needed.

We are interested in how close you feel to each person below. Compared to everyone you've listed, how close do you feel to each person?

How close do you feel to [name1]?
◯ especially close
◯ not especially close

How close do you feel to [name2]?
◯ especially close
◯ not especially close

How close do you feel to [name3]?
◯ especially close
◯ not especially close

How close do you feel to [name4]?
◯ especially close
◯ not especially close

How close do you feel to [name5]?
◯ especially close
◯ not especially close

How close do you feel to [name6]?
◯ especially close
◯ not especially close

How close do you feel to [name7]?
◯ especially close
◯ not especially close

How close do you feel to [name8]?
◯ especially close
◯ not especially close

How close do you feel to [name9]?	◯ especially close<br>◯ not especially close
How close do you feel to [name10]?	◯ especially close<br>◯ not especially close
How close do you feel to [name11]?	◯ especially close<br>◯ not especially close
How close do you feel to [name12]?	◯ especially close<br>◯ not especially close
How close do you feel to [name13]?	◯ especially close<br>◯ not especially close
How close do you feel to [name14]?	◯ especially close<br>◯ not especially close
How close do you feel to [name15]?	◯ especially close<br>◯ not especially close

You've just finished the most important part of the survey&#8212 you're 25% done!

We will now focus on the relationship between each pair of people you mentioned. For example, we will ask about [name1] and [name2]. For each pair, we will ask whether they are total strangers, in-between, or especially close. Here is what we mean by each term:

Total strangers: The two people wouldn't recognize one another if they met on the street.

In-between: Relationships in the middle of total strangers and especially close. Typically, these people are casual acquaintances.

Especially close: As close or closer to each other than they are to you.

Take your time. This can be difficult.

Is [name1] a total stranger, especially close, or in-between with [name2]?	◯ stranger<br>◯ in-between<br>◯ especially close
Is [name1] a total stranger, especially close, or in-between with [name3]?	◯ stranger<br>◯ in-between<br>◯ especially close
Is [name1] a total stranger, especially close, or in-between with [name4]?	◯ stranger<br>◯ in-between<br>◯ especially close
Is [name1] a total stranger, especially close, or in-between with [name5]?	◯ stranger<br>◯ in-between<br>◯ especially close

Is [name1] a total stranger, especially close, or in-between with [name6]?	◯ stranger<br>◯ in-between<br>◯ especially close
Is [name1] a total stranger, especially close, or in-between with [name7]?	◯ stranger<br>◯ in-between<br>◯ especially close
Is [name1] a total stranger, especially close, or in-between with [name8]?	◯ stranger<br>◯ in-between<br>◯ especially close
Is [name1] a total stranger, especially close, or in-between with [name9]?	◯ stranger<br>◯ in-between<br>◯ especially close
Is [name1] a total stranger, especially close, or in-between with [name10]?	◯ stranger<br>◯ in-between<br>◯ especially close
Is [name1] a total stranger, especially close, or in-between with [name11]?	◯ stranger<br>◯ in-between<br>◯ especially close
Is [name1] a total stranger, especially close, or in-between with [name12]?	◯ stranger<br>◯ in-between<br>◯ especially close
Is [name1] a total stranger, especially close, or in-between with [name13]?	◯ stranger<br>◯ in-between<br>◯ especially close
Is [name1] a total stranger, especially close, or in-between with [name14]?	◯ stranger<br>◯ in-between<br>◯ especially close
Is [name1] a total stranger, especially close, or in-between with [name15]?	◯ stranger<br>◯ in-between<br>◯ especially close
- - - - - - - - - - - -	
Is [name2] a total stranger, especially close, or in-between with [name3]?	◯ stranger<br>◯ in-between<br>◯ especially close
Is [name2] a total stranger, especially close, or in-between with [name4]?	◯ stranger<br>◯ in-between<br>◯ especially close
Is [name2] a total stranger, especially close, or in-between with [name5]?	◯ stranger<br>◯ in-between<br>◯ especially close
Is [name2] a total stranger, especially close, or in-between with [name6]?	◯ stranger<br>◯ in-between<br>◯ especially close

Is [name2] a total stranger, especially close, or in-between with [name7]?	◯ stranger<br>◯ in-between<br>◯ especially close
Is [name2] a total stranger, especially close, or in-between with [name8]?	◯ stranger<br>◯ in-between<br>◯ especially close
Is [name2] a total stranger, especially close, or in-between with [name9]?	◯ stranger<br>◯ in-between<br>◯ especially close
Is [name2] a total stranger, especially close, or in-between with [name10]?	◯ stranger<br>◯ in-between<br>◯ especially close
Is [name2] a total stranger, especially close, or in-between with [name11]?	◯ stranger<br>◯ in-between<br>◯ especially close
Is [name2] a total stranger, especially close, or in-between with [name12]?	◯ stranger<br>◯ in-between<br>◯ especially close
Is [name2] a total stranger, especially close, or in-between with [name13]?	◯ stranger<br>◯ in-between<br>◯ especially close
Is [name2] a total stranger, especially close, or in-between with [name14]?	◯ stranger<br>◯ in-between<br>◯ especially close
Is [name2] a total stranger, especially close, or in-between with [name15]?	◯ stranger<br>◯ in-between<br>◯ especially close
- - - - - - - - - - - - -	
Is [name3] a total stranger, especially close, or in-between with [name4]?	◯ stranger<br>◯ in-between<br>◯ especially close
Is [name3] a total stranger, especially close, or in-between with [name5]?	◯ stranger<br>◯ in-between<br>◯ especially close
Is [name3] a total stranger, especially close, or in-between with [name6]?	◯ stranger<br>◯ in-between<br>◯ especially close
Is [name3] a total stranger, especially close, or in-between with [name7]?	◯ stranger<br>◯ in-between<br>◯ especially close
Is [name3] a total stranger, especially close, or in-between with [name8]?	◯ stranger<br>◯ in-between<br>◯ especially close

Is [name3] a total stranger, especially close, or in-between with [name9]?	◯ stranger<br>◯ in-between<br>◯ especially close
Is [name3] a total stranger, especially close, or in-between with [name10]?	◯ stranger<br>◯ in-between<br>◯ especially close
Is [name3] a total stranger, especially close, or in-between with [name11]?	◯ stranger<br>◯ in-between<br>◯ especially close
Is [name3] a total stranger, especially close, or in-between with [name12]?	◯ stranger<br>◯ in-between<br>◯ especially close
Is [name3] a total stranger, especially close, or in-between with [name13]?	◯ stranger<br>◯ in-between<br>◯ especially close
Is [name3] a total stranger, especially close, or in-between with [name14]?	◯ stranger<br>◯ in-between<br>◯ especially close
Is [name3] a total stranger, especially close, or in-between with [name15]?	◯ stranger<br>◯ in-between<br>◯ especially close
- - - - - - - - - - - -	
Is [name4] a total stranger, especially close, or in-between with [name5]?	◯ stranger<br>◯ in-between<br>◯ especially close
Is [name4] a total stranger, especially close, or in-between with [name6]?	◯ stranger<br>◯ in-between<br>◯ especially close
Is [name4] a total stranger, especially close, or in-between with [name7]?	◯ stranger<br>◯ in-between<br>◯ especially close
Is [name4] a total stranger, especially close, or in-between with [name8]?	◯ stranger<br>◯ in-between<br>◯ especially close
Is [name4] a total stranger, especially close, or in-between with [name9]?	◯ stranger<br>◯ in-between<br>◯ especially close
Is [name4] a total stranger, especially close, or in-between with [name10]?	◯ stranger<br>◯ in-between<br>◯ especially close
Is [name4] a total stranger, especially close, or in-between with [name11]?	◯ stranger<br>◯ in-between<br>◯ especially close

Is [name4] a total stranger, especially close, or in-between with [name12]?	◯ stranger<br>◯ in-between<br>◯ especially close
Is [name4] a total stranger, especially close, or in-between with [name13]?	◯ stranger<br>◯ in-between<br>◯ especially close
Is [name4] a total stranger, especially close, or in-between with [name14]?	◯ stranger<br>◯ in-between<br>◯ especially close
Is [name4] a total stranger, especially close, or in-between with [name15]?	◯ stranger<br>◯ in-between<br>◯ especially close
- - - - - - - - - - - - -	
Is [name5] a total stranger, especially close, or in-between with [name6]?	◯ stranger<br>◯ in-between<br>◯ especially close
Is [name5] a total stranger, especially close, or in-between with [name7]?	◯ stranger<br>◯ in-between<br>◯ especially close
Is [name5] a total stranger, especially close, or in-between with [name8]?	◯ stranger<br>◯ in-between<br>◯ especially close
Is [name5] a total stranger, especially close, or in-between with [name9]?	◯ stranger<br>◯ in-between<br>◯ especially close
Is [name5] a total stranger, especially close, or in-between with [name10]?	◯ stranger<br>◯ in-between<br>◯ especially close
Is [name5] a total stranger, especially close, or in-between with [name11]?	◯ stranger<br>◯ in-between<br>◯ especially close
Is [name5] a total stranger, especially close, or in-between with [name12]?	◯ stranger<br>◯ in-between<br>◯ especially close
Is [name5] a total stranger, especially close, or in-between with [name13]?	◯ stranger<br>◯ in-between<br>◯ especially close
Is [name5] a total stranger, especially close, or in-between with [name14]?	◯ stranger<br>◯ in-between<br>◯ especially close
Is [name5] a total stranger, especially close, or in-between with [name15]?	◯ stranger<br>◯ in-between<br>◯ especially close

- - - - - - - - - - - -

Is [name6] a total stranger, especially close, or in-between with [name7]?	◯ stranger<br>◯ in-between<br>◯ especially close
Is [name6] a total stranger, especially close, or in-between with [name8]?	◯ stranger<br>◯ in-between<br>◯ especially close
Is [name6] a total stranger, especially close, or in-between with [name9]?	◯ stranger<br>◯ in-between<br>◯ especially close
Is [name6] a total stranger, especially close, or in-between with [name10]?	◯ stranger<br>◯ in-between<br>◯ especially close
Is [name6] a total stranger, especially close, or in-between with [name11]?	◯ stranger<br>◯ in-between<br>◯ especially close
Is [name6] a total stranger, especially close, or in-between with [name12]?	◯ stranger<br>◯ in-between<br>◯ especially close
Is [name6] a total stranger, especially close, or in-between with [name13]?	◯ stranger<br>◯ in-between<br>◯ especially close
Is [name6] a total stranger, especially close, or in-between with [name14]?	◯ stranger<br>◯ in-between<br>◯ especially close
Is [name6] a total stranger, especially close, or in-between with [name15]?	◯ stranger<br>◯ in-between<br>◯ especially close

- - - - - - - - - - - -

Is [name7] a total stranger, especially close, or in-between with [name8]?	◯ stranger<br>◯ in-between<br>◯ especially close
Is [name7] a total stranger, especially close, or in-between with [name9]?	◯ stranger<br>◯ in-between<br>◯ especially close
Is [name7] a total stranger, especially close, or in-between with [name10]?	◯ stranger<br>◯ in-between<br>◯ especially close
Is [name7] a total stranger, especially close, or in-between with [name11]?	◯ stranger<br>◯ in-between<br>◯ especially close

Is [name7] a total stranger, especially close, or in-between with [name12]?	◯ stranger<br>◯ in-between<br>◯ especially close
Is [name7] a total stranger, especially close, or in-between with [name13]?	◯ stranger<br>◯ in-between<br>◯ especially close
Is [name7] a total stranger, especially close, or in-between with [name14]?	◯ stranger<br>◯ in-between<br>◯ especially close
Is [name7] a total stranger, especially close, or in-between with [name15]?	◯ stranger<br>◯ in-between<br>◯ especially close
- - - - - - - - - - - -	
Is [name8] a total stranger, especially close, or in-between with [name9]?	◯ stranger<br>◯ in-between<br>◯ especially close
Is [name8] a total stranger, especially close, or in-between with [name10]?	◯ stranger<br>◯ in-between<br>◯ especially close
Is [name8] a total stranger, especially close, or in-between with [name11]?	◯ stranger<br>◯ in-between<br>◯ especially close
Is [name8] a total stranger, especially close, or in-between with [name12]?	◯ stranger<br>◯ in-between<br>◯ especially close
Is [name8] a total stranger, especially close, or in-between with [name13]?	◯ stranger<br>◯ in-between<br>◯ especially close
Is [name8] a total stranger, especially close, or in-between with [name14]?	◯ stranger<br>◯ in-between<br>◯ especially close
Is [name8] a total stranger, especially close, or in-between with [name15]?	◯ stranger<br>◯ in-between<br>◯ especially close
- - - - - - - - - - - -	
Is [name9] a total stranger, especially close, or in-between with [name10]?	◯ stranger<br>◯ in-between<br>◯ especially close
Is [name9] a total stranger, especially close, or in-between with [name11]?	◯ stranger<br>◯ in-between<br>◯ especially close

Is [name9] a total stranger, especially close, or in-between with [name12]?	◯ stranger<br>◯ in-between<br>◯ especially close
Is [name9] a total stranger, especially close, or in-between with [name13]?	◯ stranger<br>◯ in-between<br>◯ especially close
Is [name9] a total stranger, especially close, or in-between with [name14]?	◯ stranger<br>◯ in-between<br>◯ especially close
Is [name9] a total stranger, especially close, or in-between with [name15]?	◯ stranger<br>◯ in-between<br>◯ especially close
- - - - - - - - - - - -	
Is [name10] a total stranger, especially close, or in-between with [name11]?	◯ stranger<br>◯ in-between<br>◯ especially close
Is [name10] a total stranger, especially close, or in-between with [name12]?	◯ stranger<br>◯ in-between<br>◯ especially close
Is [name10] a total stranger, especially close, or in-between with [name13]?	◯ stranger<br>◯ in-between<br>◯ especially close
Is [name10] a total stranger, especially close, or in-between with [name14]?	◯ stranger<br>◯ in-between<br>◯ especially close
Is [name10] a total stranger, especially close, or in-between with [name15]?	◯ stranger<br>◯ in-between<br>◯ especially close
- - - - - - - - - - - -	
Is [name11] a total stranger, especially close, or in-between with [name12]?	◯ stranger<br>◯ in-between<br>◯ especially close
Is [name11] a total stranger, especially close, or in-between with [name13]?	◯ stranger<br>◯ in-between<br>◯ especially close
Is [name11] a total stranger, especially close, or in-between with [name14]?	◯ stranger<br>◯ in-between<br>◯ especially close
Is [name11] a total stranger, especially close, or in-between with [name15]?	◯ stranger<br>◯ in-between<br>◯ especially close
- - - - - - - - - - - -	

Is [name12] a total stranger, especially close, or in-between with [name13]?	◯ stranger<br>◯ in-between<br>◯ especially close
Is [name12] a total stranger, especially close, or in-between with [name14]?	◯ stranger<br>◯ in-between<br>◯ especially close
Is [name12] a total stranger, especially close, or in-between with [name15]?	◯ stranger<br>◯ in-between<br>◯ especially close
- - - - - - - - - - - -	
Is [name13] a total stranger, especially close, or in-between with [name14]?	◯ stranger<br>◯ in-between<br>◯ especially close
Is [name13] a total stranger, especially close, or in-between with [name15]?	◯ stranger<br>◯ in-between<br>◯ especially close
- - - - - - - - - - - -	
Is [name14] a total stranger, especially close, or in-between with [name15]?	◯ stranger<br>◯ in-between<br>◯ especially close
You've just completed the hardest part&#8212 keep going!	

About People in Your Network

We are going to ask you a series of questions about each person in your network. In some cases, you may not know the exact answer for sure. Whenever possible, we would appreciate if you would guess - try to use the "don't know" category as little as possible.

Which person or persons support you most often? You can choose more than one person.

	Supports me most often
[name1]	◯
[name2]	◯
[name3]	◯
[name4]	◯
[name5]	◯
[name6]	◯
[name7]	◯
[name8]	◯

[name9]	◯
[name10]	◯
[name11]	◯
[name12]	◯
[name13]	◯
[name14]	◯
[name15]	◯

We are going to ask you about what types of support each person offers you. These are the ways that we categorize each type of support:

Emotional Support: This would be someone who you turn to when you need help managing personal situations, and share feelings of happiness or sadness.Life Advice: This would be someone who gives you advice on life decisions such as employment or places to live.Financial Support: This would be someone who gives you advice or provides direct help with your daily or overall finances.Physical or Health Support: This would be someone who gives you advice on or provides direct help with medications/supplements, choosing doctors or getting to appointments, mental health resources, managing health insurance, or physical assistance around your household.Camaraderie: This would be someone who provides support through understanding important experiences that you have had together.
What kind of support does each person give you? (Select all that apply)

	Emotional support	Life advice	Financial support	Physical or Health support	Camaraderie	None
[name1]	☐	☐	☐	☐	☐	☐
[name2]	☐	☐	☐	☐	☐	☐
[name3]	☐	☐	☐	☐	☐	☐
[name4]	☐	☐	☐	☐	☐	☐
[name5]	☐	☐	☐	☐	☐	☐
[name6]	☐	☐	☐	☐	☐	☐
[name7]	☐	☐	☐	☐	☐	☐
[name8]	☐	☐	☐	☐	☐	☐
[name9]	☐	☐	☐	☐	☐	☐
[name10]	☐	☐	☐	☐	☐	☐
[name11]	☐	☐	☐	☐	☐	☐
[name12]	☐	☐	☐	☐	☐	☐
[name13]	☐	☐	☐	☐	☐	☐
[name14]	☐	☐	☐	☐	☐	☐
[name15]	☐	☐	☐	☐	☐	☐

What sex is each person in your network?

	Male	Female
[name1]	○	○
[name2]	○	○
[name3]	○	○
[name4]	○	○
[name5]	○	○
[name6]	○	○
[name7]	○	○
[name8]	○	○
[name9]	○	○
[name10]	○	○
[name11]	○	○
[name12]	○	○
[name13]	○	○
[name14]	○	○
[name15]	○	○

Do any people in your network have a negative influence on your health? For example, some people can passively or actively encourage you to smoke, not eat well, or not exercise.

[name1]	○ Yes<br>○ No
[name2]	○ Yes<br>○ No
[name3]	○ Yes<br>○ No
[name4]	○ Yes<br>○ No
[name5]	○ Yes<br>○ No
[name6]	○ Yes<br>○ No
[name7]	○ Yes<br>○ No
[name8]	○ Yes<br>○ No
[name9]	○ Yes<br>○ No
[name10]	○ Yes<br>○ No

[name11]	◯ Yes<br>◯ No
[name12]	◯ Yes<br>◯ No
[name13]	◯ Yes<br>◯ No
[name14]	◯ Yes<br>◯ No
[name15]	◯ Yes<br>◯ No

What is the race of each person in your social network? (Select all that apply)

	Black or African American	White	American Indian/Alaska Native	Asian	Native Hawaiian or Other Pacific Islander	Other	Don't know
[name1]	☐	☐	☐	☐	☐	☐	☐
[name2]	☐	☐	☐	☐	☐	☐	☐
[name3]	☐	☐	☐	☐	☐	☐	☐
[name4]	☐	☐	☐	☐	☐	☐	☐
[name5]	☐	☐	☐	☐	☐	☐	☐
[name6]	☐	☐	☐	☐	☐	☐	☐
[name7]	☐	☐	☐	☐	☐	☐	☐
[name8]	☐	☐	☐	☐	☐	☐	☐
[name9]	☐	☐	☐	☐	☐	☐	☐
[name10]	☐	☐	☐	☐	☐	☐	☐
[name11]	☐	☐	☐	☐	☐	☐	☐
[name12]	☐	☐	☐	☐	☐	☐	☐
[name13]	☐	☐	☐	☐	☐	☐	☐
[name14]	☐	☐	☐	☐	☐	☐	☐
[name15]	☐	☐	☐	☐	☐	☐	☐

As far as you know, what is the highest level of education of each person?

	Some high school or less	High school grad	Some college	Associate degree	Bachelor's degree	Graduate degree	Don't know
[name1]							
[name2]							
[name3]							
[name4]							
[name5]							
[name6]							
[name7]							
[name8]							
[name9]							
[name10]							
[name11]							
[name12]							
[name13]							
[name14]							
[name15]							

On average, how often do you communicate with each person in your network?

	Daily	Weekly	Monthly	Less often	Don't know
[name1]					
[name2]					
[name3]					
[name4]					
[name5]					
[name6]					
[name7]					
[name8]					
[name9]					
[name10]					
[name11]					
[name12]					
[name13]					
[name14]					
[name15]					

For how many years have you known the following people?

	Less than three	Three to six	More than six	Don't know
[name1]	○	○	○	○
[name2]	○	○	○	○
[name3]	○	○	○	○
[name4]	○	○	○	○
[name5]	○	○	○	○
[name6]	○	○	○	○
[name7]	○	○	○	○
[name8]	○	○	○	○
[name9]	○	○	○	○
[name10]	○	○	○	○
[name11]	○	○	○	○
[name12]	○	○	○	○
[name13]	○	○	○	○
[name14]	○	○	○	○
[name15]	○	○	○	○

We are now going to ask all the ways each person is connected to you. Some people can be connected to you in more than one way. For example, a man could be your brother, and he could belong to your church and be your lawyer.

For each person below, in what way are they connected to you? (Select all that apply)

	Spouse	Family	Friend	Advisor	Co - worker	Other
[name1]	☐	☐	☐	☐	☐	☐
[name2]	☐	☐	☐	☐	☐	☐
[name3]	☐	☐	☐	☐	☐	☐
[name4]	☐	☐	☐	☐	☐	☐
[name5]	☐	☐	☐	☐	☐	☐
[name6]	☐	☐	☐	☐	☐	☐
[name7]	☐	☐	☐	☐	☐	☐
[name8]	☐	☐	☐	☐	☐	☐
[name9]	☐	☐	☐	☐	☐	☐
[name10]	☐	☐	☐	☐	☐	☐
[name11]	☐	☐	☐	☐	☐	☐
[name12]	☐	☐	☐	☐	☐	☐
[name13]	☐	☐	☐	☐	☐	☐
[name14]	☐	☐	☐	☐	☐	☐
[name15]	☐	☐	☐	☐	☐	☐

Did anyone in your network play football? (Select the highest level in which they competed)

	High school football	College football	Professional football	Not a football player
[name1]	○	○	○	○
[name2]	○	○	○	○
[name3]	○	○	○	○
[name4]	○	○	○	○
[name5]	○	○	○	○
[name6]	○	○	○	○
[name7]	○	○	○	○
[name8]	○	○	○	○
[name9]	○	○	○	○
[name10]	○	○	○	○
[name11]	○	○	○	○
[name12]	○	○	○	○
[name13]	○	○	○	○
[name14]	○	○	○	○
[name15]	○	○	○	○

Did anyone in your network play with you or coach you during your football career? (Select all that apply)

	Former Professional Teammate	Former Professional Coach	Former HS or College Teammate	Former HS or College Coach	Other Football connection	No football connection
[name1]	☐	☐	☐	☐	☐	☐
[name2]	☐	☐	☐	☐	☐	☐
[name3]	☐	☐	☐	☐	☐	☐
[name4]	☐	☐	☐	☐	☐	☐
[name5]	☐	☐	☐	☐	☐	☐
[name6]	☐	☐	☐	☐	☐	☐
[name7]	☐	☐	☐	☐	☐	☐
[name8]	☐	☐	☐	☐	☐	☐
[name9]	☐	☐	☐	☐	☐	☐
[name10]	☐	☐	☐	☐	☐	☐
[name11]	☐	☐	☐	☐	☐	☐
[name12]	☐	☐	☐	☐	☐	☐
[name13]	☐	☐	☐	☐	☐	☐
[name14]	☐	☐	☐	☐	☐	☐
[name15]	☐	☐	☐	☐	☐	☐

How old is each person? If you don't know the exact age, your best guess is ok.	
[name1]	______________________
[name2]	______________________
[name3]	______________________
[name4]	______________________
[name5]	______________________
[name6]	______________________
[name7]	______________________
[name8]	______________________
[name9]	______________________
[name10]	______________________
[name11]	______________________
[name12]	______________________
[name13]	______________________
[name14]	______________________
[name15]	______________________

You've entered the survey's Red Zone! Almost Done!

Health Habits

Please remember that the more carefully you can answer the following questions about yourself and those in your network, the more you are helping the research, and in turn helping your fellow teammates.

Have you cut back on heavy drinking of alcohol in the past 3 months?	◯ Yes<br>◯ No<br>◯ I do not drink heavily
Have you cut back on performance enhancing drugs or supplements in the past 3 months?	◯ Yes<br>◯ No<br>◯ I do not use performance enhancing drugs or supplements
Have you exercised at least 3-4 times a week over the past 3 months?	◯ Yes<br>◯ No
Did you eat a healthy diet regularly over the past 3 months?	◯ Yes<br>◯ No
In which areas do you feel you experience more health problems than the average person? (Select all that apply)	☐ General Health<br>☐ Pain<br>☐ Cognitive/Mental Health<br>☐ Cardiac<br>☐ No Health Problems

Which people in your network do you think have cut back on heavy drinking of alcohol in the past 3 months?

	Yes	No	Does not drink heavily	Don't know
[name1]	◯	◯	◯	◯
[name2]	◯	◯	◯	◯
[name3]	◯	◯	◯	◯
[name4]	◯	◯	◯	◯
[name5]	◯	◯	◯	◯
[name6]	◯	◯	◯	◯
[name7]	◯	◯	◯	◯
[name8]	◯	◯	◯	◯
[name9]	◯	◯	◯	◯
[name10]	◯	◯	◯	◯
[name11]	◯	◯	◯	◯
[name12]	◯	◯	◯	◯
[name13]	◯	◯	◯	◯
[name14]	◯	◯	◯	◯
[name15]	◯	◯	◯	◯

Which people in your network do you think have cut back on performance enhancing drugs or supplements in the past 3 months?

	Yes	No	Does not take performance enhancing drugs or supplements	Don't know
[name1]	○	○	○	○
[name2]	○	○	○	○
[name3]	○	○	○	○
[name4]	○	○	○	○
[name5]	○	○	○	○
[name6]	○	○	○	○
[name7]	○	○	○	○
[name8]	○	○	○	○
[name9]	○	○	○	○
[name10]	○	○	○	○
[name11]	○	○	○	○
[name12]	○	○	○	○
[name13]	○	○	○	○
[name14]	○	○	○	○
[name15]	○	○	○	○

Which people in your network do you think have exercised at least 3-4 times a week in the past 3 months?

	Yes	No	Don't know
[name1]	○	○	○
[name2]	○	○	○
[name3]	○	○	○
[name4]	○	○	○
[name5]	○	○	○
[name6]	○	○	○
[name7]	○	○	○
[name8]	○	○	○
[name9]	○	○	○
[name10]	○	○	○
[name11]	○	○	○
[name12]	○	○	○
[name13]	○	○	○
[name14]	○	○	○
[name15]	○	○	○

Which people in your network do you think have eaten a healthy diet regularly over the past 3 months?

	Yes	No	Don't know
[name1]	◯	◯	◯
[name2]	◯	◯	◯
[name3]	◯	◯	◯
[name4]	◯	◯	◯
[name5]	◯	◯	◯
[name6]	◯	◯	◯
[name7]	◯	◯	◯
[name8]	◯	◯	◯
[name9]	◯	◯	◯
[name10]	◯	◯	◯
[name11]	◯	◯	◯
[name12]	◯	◯	◯
[name13]	◯	◯	◯
[name14]	◯	◯	◯
[name15]	◯	◯	◯

In which areas do people in your network experience health problems that are more than typical for an average person? (Select all that apply)

	General Health	Pain	Cognitive/Mental Health	Cardiac	No Health Problems	Don't know
[name1]	☐	☐	☐	☐	☐	☐
[name2]	☐	☐	☐	☐	☐	☐
[name3]	☐	☐	☐	☐	☐	☐
[name4]	☐	☐	☐	☐	☐	☐
[name5]	☐	☐	☐	☐	☐	☐
[name6]	☐	☐	☐	☐	☐	☐
[name7]	☐	☐	☐	☐	☐	☐
[name8]	☐	☐	☐	☐	☐	☐
[name9]	☐	☐	☐	☐	☐	☐
[name10]	☐	☐	☐	☐	☐	☐
[name11]	☐	☐	☐	☐	☐	☐
[name12]	☐	☐	☐	☐	☐	☐
[name13]	☐	☐	☐	☐	☐	☐
[name14]	☐	☐	☐	☐	☐	☐
[name15]	☐	☐	☐	☐	☐	☐

How far does each person live from you?

	Same house	0-5 miles	6-15 miles	16-50 miles	50+ miles
[name1]	◯	◯	◯	◯	◯
[name2]	◯	◯	◯	◯	◯
[name3]	◯	◯	◯	◯	◯
[name4]	◯	◯	◯	◯	◯
[name5]	◯	◯	◯	◯	◯
[name6]	◯	◯	◯	◯	◯
[name7]	◯	◯	◯	◯	◯
[name8]	◯	◯	◯	◯	◯
[name9]	◯	◯	◯	◯	◯
[name10]	◯	◯	◯	◯	◯
[name11]	◯	◯	◯	◯	◯
[name12]	◯	◯	◯	◯	◯
[name13]	◯	◯	◯	◯	◯
[name14]	◯	◯	◯	◯	◯
[name15]	◯	◯	◯	◯	◯

Your Football History

Which NFL team do you primarily identify with?

◯ 49ers
◯ Bears
◯ Bengals
◯ Bills
◯ Broncos
◯ Browns
◯ Buccaneers
◯ Cardinals
◯ Chargers
◯ Chiefs
◯ Colts
◯ Cowboys
◯ Dolphins
◯ Eagles
◯ Falcons
◯ Giants
◯ Jaguars
◯ Jets
◯ Lions
◯ Packers
◯ Panthers
◯ Patriots
◯ Raiders
◯ Rams
◯ Ravens
◯ Redskins
◯ Saints
◯ Seahawks
◯ Steelers
◯ Texans
◯ Titans
◯ Vikings

(This is not necessarily the team for which you played the longest.)

How many years did you play for the team selected above?

____

How many different NFL teams did you play with during your career?	◯ 1<br>◯ 2<br>◯ 3<br>◯ 4<br>◯ 5<br>◯ 6<br>◯ 7<br>◯ 8<br>◯ 9<br>◯ 10<br>◯ 11<br>◯ 12<br>◯ 13<br>◯ 14<br>◯ 15<br>◯ 16<br>◯ 17<br>◯ 18<br>◯ 19<br>◯ 20<br>◯ 21<br>◯ 22<br>◯ 23<br>◯ 24<br>◯ 25<br>◯ 26<br>◯ 27<br>◯ 28<br>◯ 29<br>◯ 30<br>◯ 31<br>◯ 32

Finally...

Did you encounter any technical difficulties during this survey?	◯ Yes<br>◯ No
You have provided important information regarding the personal networks of athletes. We are interested in seeing how personal networks change over time. Would you be interested in being contacted in the future for a follow up study?	◯ Yes<br>◯ No
How did you hear about the personal network survey? (Select all that apply)	☐ Email from the Football Players Health Study<br>☐ Football Players Health Study Social Media<br>☐ From a friend who is a former player<br>☐ From a spouse/partner<br>☐ Other
Please specify:	________________________________

Supplementary Methods 2: Network Diagram and Information from Experts

Example Network Diagram Received by Participants

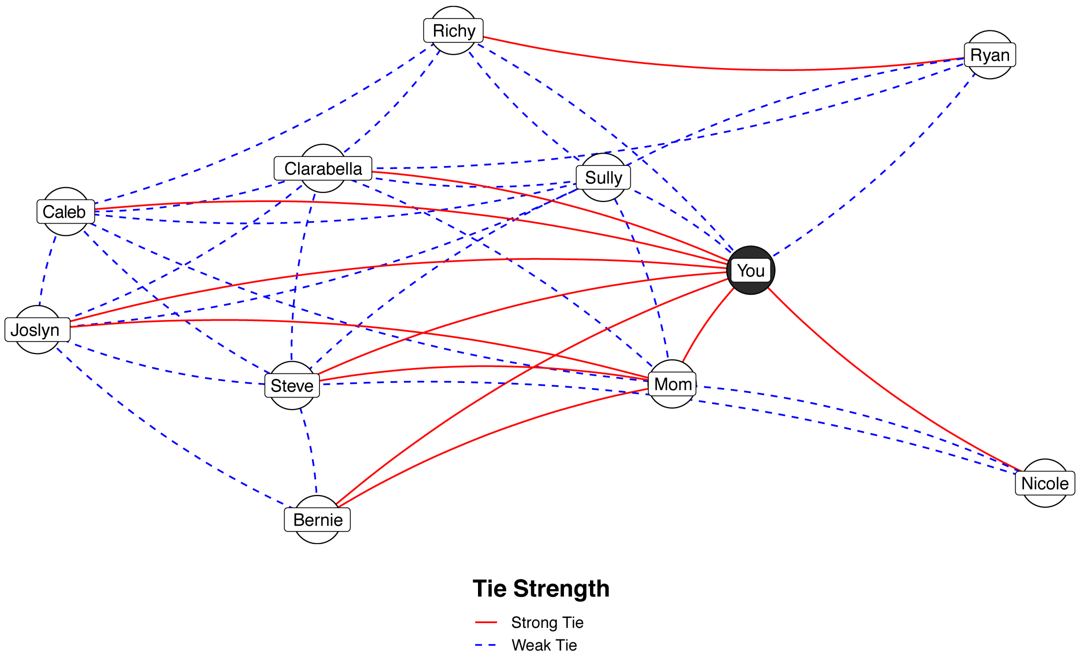

Social Network Characteristics	Your Network
Size of your network	10 People
Density of ties in your network	66.7%
Percent who are family	50%
Percent who are teammates	20%
Percent who eat a healthy diet	30%
Percent who exercise regularly	30%
Percent who drink alcohol heavily	40%
Percent who take performance enhancing drugs or supplements	60%
Percent who have health problems	50%

Example “From the Experts” File received by Participants

THE FOOTBALL PLAYERS
HEALTH STUDY
AT HARVARD UNIVERSITY

Thank you so much for participating in the Personal Network Survey with the Harvard Football Players Health Study.

At this point you may be asking…

“What is this personal network image I’ve received?”
“Why is the information in the table important?”
“How do I apply this information to my daily life?”

We’ve compiled some expert feedback from a panel of doctors to address those exact questions.

Read on to better understand why this research is relevant to your health, and how you can best utilize your personal results.

1. How do you know that my personal network is important to my health?

“**There is a large body of scientific evidence suggesting that an individual’s personal network is related to whether you develop acute or chronic health conditions, and the extent and speed of recovery from illness.** Personal network characteristics have been linked to risk for depression, anxiety, alcoholism, and drug abuse, as well as general feelings of psychological distress. Additionally, cardiovascular disease, hypertension, infectious disease, aging-related dementia, and cancer survival have all been linked to personal networks. All in all, personal networks and relationships are among the strongest and most robust predictors of health outcomes among all the factors studied by health researchers.

Personal networks help you cope with stressful conditions and negative life events, reducing the harmful effects of stress on the body. Members of your personal network can also influence your behavior, for better or worse. Some networks encourage positive behavior like exercising regularly, eating a healthy diet, or seeking health services, while others can affect behavior in negative ways. Personal networks are also a source of support resources that can reduce your odds of getting sick and promote recovery when illness does occur. Resources come in many forms, including emotional support (e.g., listening to your problems), informational support (e.g., referring you to a good doctor), financial support (e.g., loaning you money), or instrumental support (e.g., giving you a ride to the hospital).”

“Scientists have been interested in the impact of relationships on health for over 200 years. Initially, their interest had to do with how marriage affects people’s health. Over the last 50 years they have broadened their scope to also consider how other relationships - parents, children, friends - affect our health. **The way scientists do these studies is that they assemble groups of people who they follow: they measure their relationships today, and then they follow their health outcomes over time.** By doing statistical analyses they’re able to tell the difference in health outcomes between those who have different amounts and types of social connections.”

“As humans have evolved to seek connection with others, there are some basic ways in which we feel better when we are around other people. **Research highlights[1,2] that those who have stronger networks of friends and family tend to be healthier, and more able to deal with health issues that arise.** Also, when your behavior or your health changes you may not see those changes in yourself, but if you have people in your personal network they might be able to identify them and step forward to support you.”

[1] House, J.S., Landis, K.R. and Umberson, D., 1988. Social relationships and health. *Science*, *241*(4865), pp.540-545.

[2] Steptoe A, Shankar A, Demakakos P, Wardle J., 2013. Social isolation, loneliness, and all-cause mortality in older men and women. *Proceedings of the National Academy of Sciences*, *110*(15), pp.5797-5801.

2. Why is the size of my network important?

"In most cases, having a large network is associated with better health outcomes. Network size is an indication of social integration, or the degree to which you are connected to others in your group or community. **People who are socially integrated feel a sense of belonging and purpose that is protective of psychological health and encourages healthy and prosocial behavior.** Network diversity may also reflect access to a wealth of support resources. The more different individuals you interact with on a regular basis, the more likely you are to be able to get your needs met through social support. Of course, in some cases having a large network can have negative consequences, as when your relationships are stressful or burdensome, or peers encourage unhealthy behavior."

"**The size of the network, meaning the number of people in it, is the basic building block of the personal network. It can be thought of as the number of life-lines in the immediate social environment.** Typically, a large network has benefits of providing more options for support, information, and practical resources. When a person is embedded in a large network, there is a greater chance for a diversity of opinions and ideas to circulate. However, maintaining a large network usually takes more effort, which is more difficult when an individual becomes sick, moves, or has a major life event. In those instances, a small network may be less draining. So, it depends on your life situation, but usually, larger is better for health."

"Just like in football - size matters! But it's not the case that one particular size is best for everyone. Sometimes a small network has advantages that a big network doesn't, and vice versa - certain sizes are related to certain kinds of health outcomes. **Each person has a unique network and it's more interesting to think about the potential mismatch between the kind of network the person has and the kind of health needs that they have.**"

3. What does "density of ties" mean, and why is it important?

"**The density of your network is the degree to which your network members have relationships with one another.** Density may reflect personal interaction styles. Some people prefer to be in a social circle where everyone knows everyone else, and they tend to introduce friends and family to each other. Others enjoy interacting one-on-one or in smaller groups, and prefer to keep their relationships separate. Alternatively, density may be an indication of social opportunity or range. People who move in many different social circles, who are particularly socially active, or who have high social status tend to have more opportunities to meet and form relationships with others who do not know one another.

Density can be associated with better or worse health, depending on what kinds of social processes are beneficial for a particular outcome. High density is often indicative of greater similarity, more trust, and stronger social bonds. These kinds of networks provide an excellent safety net in times of trouble. However, low density networks can also be beneficial. They are associated with access to novel information and diverse resources."

"For an individual person, "density of ties" means how well the people in your network know each other. To what extent are you the center of a set of relationships where no one else knows each other? If that's the case, it means you have a lot of different people you might turn to who aren't connected to each other, and if something difficult happens in one particular relationship it won't ripple through your network. **If people in your network aren't closely connected to each other they might be more likely to know different things and have more versatile ways in which they can help you. On the other hand, it may mean you don't do things with people as a group, or if you need coordination amongst your network (e.g. if someone gets sick and a group of people need to coordinate to support them), it's unlikely to happen easily when the network is more disconnected.** Alternatively, if you are a part of a very closely connected network of people (a dense network), but you don't have many ties outside of that, it may mean that group of people has a very narrow picture of the world – they all know the same things, have the same perspective – and your network may not be very rich in diverse information."

4. What if I don't have a personal network? What are the implications for my health?

"If you don't have a personal network, you are in a position where you're more likely to be isolated and disconnected. This becomes important to consider because network ties can help you in a lot of ways. **People in your network can help you to know whether things that are happening to you are things that happen to everyone else, or whether they're unusual.** The network around you will also provide information about things that you might want to do or might need – like support services or social activities. Research also shows that the more connected people are, the more that they enjoy their retirement.[3]"

"**Scientists have shown that being socially isolated - not having even *one* person that you feel comfortable discussing important issues with - is a greater risk factor for health outcomes than smoking[4].** Being socially isolated is devastating for a person's health and sadly, about 5-6% of Americans are in that situation."

"If you don't have a personal network you are socially isolated and at high risk for mental and physical health problems. One study found that the size of the effect of social isolation on mortality is comparable to clinical risk factors like obesity, smoking, high blood pressure, and high cholesterol that are the major causes of premature death in the US. Similarly, feelings of loneliness are predictive of mental illness, suicide, substance abuse, dementia, and other conditions. If you feel lonely or are isolated, it is critical to become more social integrated and active. **There are many ways to achieve a fuller social life, such as joining a club, church, or other voluntary organization, or by reinvesting and becoming more engaged in lapsed friendships or family relationships**."

[3] Mor-Barak, M.E., Scharlach, A.E., Birba, L. and Sokolov, J., 1992. Employment, social networks, and health in the retirement years. *The International Journal of Aging and Human Development*, *35*(2), pp.145-159.

[4] Holt-Lunstad, J., Smith, T.B. and Layton, J.B., 2010. Social relationships and mortality risk: a meta-analytic review. *PLoS medicine*, *7*(7), p.e1000316.

5. What should I look for in my personal network?

"Here's a good exercise – list the things that you need and like in your life that potentially involve other people. Are there certain kinds of activities that you do for fun? Most activities tend to involve other people, or are more enjoyable if they involve other people. Frequently, if you share experiences with others, you actually gain more from the experience yourself. But there are also other things to consider that are more practical – do you need someone to watch your pet, or pick up your kids in a pinch? Try to have people in your network who are not just companions for activities, but also can help you with the practical elements of life – "who do I call when _____ happens?" **Think about the kind of things that you need on a day to day or even annual basis, and where it would be helpful to be able to rely on someone in your network. Human beings are wired to feel better if they help someone else. It's good to go through that kind of checklist and if you're missing something, then think about the way you lead your life and where your connections come from. Choose potential opportunities and experiences that will help you build new relationships** (e.g. joining a community group, an activity league, a new religious affiliation, etc.). Those opportunities differ for each individual, but if you identify a kind of support that is missing in your life, the question becomes how you can adjust your life to create opportunities to fill those gaps."

"We think about personal networks as being the support that's provided to you, but you can also consider how alike you are with people in your network. People who are like you are more likely to share your interests and values. If want to increase the representation of people like you in your network, you can consider places in your community you could go to meet those people. You can also think about things you want to experience in your life, and try to connect with people who can help you achieve them. For example, if you are a senior who wants to improve computer literacy, try to connect with younger groups of people who could support you in that. Do you love movies? Seek out organized movie groups nearby. **Reviewing your network is a good time to strike out and look to community resources that are available.**"

"**It's not one size fits all.** You might prefer to invest in being closer to a smaller number of friends, or you might be a social butterfly with a lot of friends. If we each have 4 friends, some of those friends might have a lot of *other* friends, but some might not. In large networks this can be quantified by "measures of centrality". The reason it's important is if you have 4 friends who have many other friends, they might not have much time for you because it is divided between many other relationships. But, they might be more connected and better able to offer information that's helpful to you - where a cool club is, or who is a good doctor for you. Alternatively, if your friends don't have many other friends, you might get more attention from them - however they'd be less able to give you access to a wider variety of information that could help you."

6. What should I consider if more of my network is made up of family vs. friends, or vice versa?

"Some research suggests that family and friends serve different functions. Family are among our longest standing, most supportive, and most dependable ties. Strong social norms influence family members to help one another and stick together, which can be especially useful during a health crisis. Conversely, we tend to seek companionship and engage in social activities more so with friends than with family, which is also important for health. **Other research indicates that the distinction between friendship and kinship has become less important over time. So-called "fictive kin" –** the term social scientists give to kinship-like bonds that are not based on blood or marriage **– are indistinguishable from traditional kin in their effects on health.** What is critical is the nature of the relationship and the social resources that are exchanged."

"**There is no one size fits all for what a 'good' network is.** It depends a lot on who you know, how they know each other, and the opportunities you have to connect to people. If you have family who are supportive in certain ways, maybe you don't need that specific type of support from your friends, or vice versa. How different types of networks manifest for different people is...different!"

"It's important to have family members in your network because they feel a real responsibility to you, whereas sometimes your friends have their own families to attend to. However, it's also good to have a mix – you might not share any interests with family, but with friends you do! You might not call on certain friends for health support, but maybe you do activities with them that enrich your life's experiences. **Diversity in networks has shown to support filling people's needs on a broader scale.**"

Meet the Experts

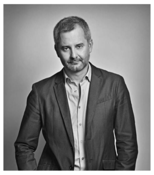

Albert-László Barabási, PhD, is the Robert Gray Dodge Professor of Network Science and a Distinguished University Professor at Northeastern University, where he directs the Center for Complex Network Research, and holds appointments in the Departments of Physics and College of Computer and Information Science, as well as in the Department of Medicine at Harvard Medical School and Brigham and Women Hospital in the Channing Division of Network Science, and is a member of the Center for Cancer Systems Biology at Dana Farber Cancer Institute. A Hungarian born native of Transylvania, Romania, he received his Masters in Theoretical Physics at the Eötvös University in Budapest, Hungary and was awarded a Ph.D. three years later at Boston University.

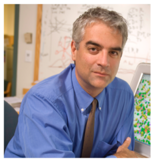

Nicholas A. Christakis, MD, PhD, MPH, is a social scientist and physician at Yale University who conducts research in the fields of network science, biosocial science, and behavior genetics. His current work focuses on how human biology and health affect, and are affected by, social interactions and social networks. He directs the Human Nature Lab and is the Co-Director of the Yale Institute for Network Science. He is the Sterling Professor of Social and Natural Science at Yale University, appointed in the Departments of Sociology; Medicine; Ecology and Evolutionary Biology; Biomedical Engineering; and the School of Management.

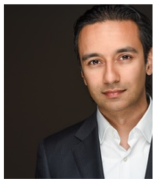

Amar Dhand, MD, DPhil, is a neurologist and network scientist. He received his MD from Harvard Medical School and his DPhil from Oxford University as a Rhodes Scholar. He completed a medical internship, neurology residency and fellowship at UCSF. He is currently Assistant Professor of Neurology at Harvard Medical School with a joint appointment at the Network Science Institute at Northeastern University. Dr. Dhand is passionate about the impact of social networks on human disease. He combines expertise in clinical neurology and social sciences for this pursuit. His lab is investigating the impact of networks on neurological health. This includes the effect of social support networks and hospital networks on patient-oriented outcomes, and the use of new tools such as social sensors.

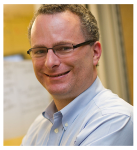

David Lazer, PhD, is a Professor of Political Science and Computer and Information Science at Northeastern University, and co-director for the NULab for Texts, Maps, and Networks. Prior to joining Northeastern University, he was on the faculty at the Harvard Kennedy School (1998-2009). His research focuses on the nexus of network science, computational social science, and collaborative intelligence. He is the founder of the citizen science website Volunteer Science and the political visualization website VisPolics. His research has been published in such journals as Science, Proceedings of the National Academy of Science, the American Political Science Review, and the Administrative Science Quarterly, and has received extensive coverage in the media, including the New York Times, NPR, the Washington Post, the Wall Street Journal, and CBS Evening News.

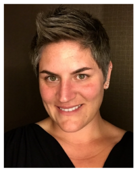

Brea L. Perry is Professor of Sociology and an affiliated faculty of the Indiana University Network Science Institute. She returned to Indiana University in 2014 after receiving her PhD at Indiana University in 2008, and then serving on the faculty at the University of Kentucky. Her research focuses on the intersections of medical sociology, biosociology, and social networks.
Dr. Perry's recent research has examined the interrelated roles of dynamic social networks, peer and family relationships, social inequality, and biological systems in disease etiology and the illness career. Much of her work examines how and why social network structure and function evolve in response to the onset of mental illness, focusing on stigma and the activation of supportive ties. Brea Perry has published her research in journals such as American Journal of Sociology, American Sociological Review, Journal of Health and Social Behavior, and Social Science and Medicine.

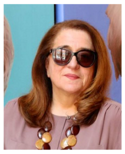

Bernice A. Pescosolido, PhD, is Distinguished and Chancellor's Professor of Sociology at Indiana University and Founding Director of the Indiana Consortium for Mental Health Services Research (ICMHSR) and the Indiana University Network Science Institute (IUNI). Her research focuses on four areas – stigma, health care use, suicide, and social networks – primarily looking at mental illness and substance abuse and the role that social and organizational networks play in people's responses to problems. Trained as a medical sociologist at Yale, her research has been published in sociology, anthropology, public health, and psychiatric journals and has been supported by the National Institute of Mental Health, the Fogarty International Center, the National Institute of Drug Abuse, the MacArthur Foundation, the Robert Wood Johnson Foundation, and the National Science Foundation, among others. She has served as the Vice President of the American Sociological Association, received several career, teaching, and mentoring awards in sociology and public health and, in 2016, was elected to the National Academy of Medicine..

Supplementary Methods 3: Equations for Social Network Metrics

$$\text{Size} = N$$

where N is the number of nodes, not including the ego.

$$\text{Density} = \frac{2L}{N(N-1)}$$

where L is the number of ties, and N is the number of nodes, not including the ego.

$$\text{Constraint of i's network} = (p_{ij} + \sum_q p_{iq} \times p_{qj})^2$$

where i is the ego, q and j are alters, p_{ij} is the proportional strength of i's relation with j, p_{iq} is the proportional strength of i's relation with q, and p_{qj} is the proportional strength of q's relation with j.

$$\text{Effective size of } i'\text{s network } = \sum_j \left[1 - \sum_q p_{iq} \times m_{jq} \right], q \neq i, j$$

where i is the ego, q and j are alters, and $\sum_q p_{iq} \times m_{jq}$ measures the portion of i's relationship with j that is redundant to i's relationships with other primary contacts.

$$\text{Maximum Degree} = L_{\max}$$

where $L_{\max}$ is the highest number of ties incident on a single node, not including the ego.

$$\text{Mean Degree} = \frac{L_1 + L_2 + \cdots + L_N}{N}$$

where L is the number of ties from each node and N is the number of nodes, not including the ego.

STROBE (Strengthening The Reporting of OBservational Studies in Epidemiology) Checklist

A checklist of items that should be included in reports of observational studies. You must report the page number in your manuscript where you consider each of the items listed in this checklist. If you have not included this information, either revise your manuscript accordingly before submitting or note N/A.

Note: An Explanation and Elaboration article discusses each checklist item and gives methodological background and published examples of transparent reporting. The STROBE checklist is best used in conjunction with this article (freely available on the Web sites of PLoS Medicine at http://www.plosmedicine.org/, Annals of Internal Medicine at http://www.annals.org/, and Epidemiology at http://www.epidem.com/). Information on the STROBE Initiative is available at www.strobe-statement.org.

Section and Item	Item No.	Recommendation	Reported on Page No.
**Title and Abstract**	1	(*a*) Indicate the study's design with a commonly used term in the title or the abstract	2
		(*b*) Provide in the abstract an informative and balanced summary of what was done and what was found	2
**Introduction**			
Background/Rationale	2	Explain the scientific background and rationale for the investigation being reported	3
Objectives	3	State specific objectives, including any prespecified hypotheses	4
**Methods**			
Study Design	4	Present key elements of study design early in the paper	4
Setting	5	Describe the setting, locations, and relevant dates, including periods of recruitment, exposure, follow-up, and data collection	12
Participants	6	(*a*) *Cohort study*—Give the eligibility criteria, and the sources and methods of selection of participants. Describe methods of follow-up	
		Case-control study—Give the eligibility criteria, and the sources and methods of case ascertainment and control selection. Give the rationale for the choice of cases and controls	
		Cross-sectional study—Give the eligibility criteria, and the sources and methods of selection of participants	12
		(*b*) *Cohort study*—For matched studies, give matching criteria and number of exposed and unexposed	
		Case-control study—For matched studies, give matching criteria and the number of controls per case	
Variables	7	Clearly define all outcomes, exposures, predictors, potential confounders, and effect modifiers. Give diagnostic criteria, if applicable	14-17

Section and Item	Item No.	Recommendation	Reported on Page No.
Data Sources/ Measurement	8*	For each variable of interest, give sources of data and details of methods of assessment (measurement). Describe comparability of assessment methods if there is more than one group	12
Bias	9	Describe any efforts to address potential sources of bias	17
Study Size	10	Explain how the study size was arrived at	12
Quantitative Variables	11	Explain how quantitative variables were handled in the analyses. If applicable, describe which groupings were chosen and why	15
Statistical Methods	12	(*a*) Describe all statistical methods, including those used to control for confounding	16
		(*b*) Describe any methods used to examine subgroups and interactions	16
		(*c*) Explain how missing data were addressed	12
		(*d*) *Cohort study*—If applicable, explain how loss to follow-up was addressed	
		Case-control study—If applicable, explain how matching of cases and controls was addressed	
		Cross-sectional study—If applicable, describe analytical methods taking account of sampling strategy	12
		(*e*) Describe any sensitivity analyses	17
**Results**			
Participants	13*	(a) Report numbers of individuals at each stage of study—eg numbers potentially eligible, examined for eligibility, confirmed eligible, included in the study, completing follow-up, and analysed	12
		(b) Give reasons for non-participation at each stage	12
		(c) Consider use of a flow diagram	
Descriptive Data	14*	(a) Give characteristics of study participants (eg demographic, clinical, social) and information on exposures and potential confounders	5
		(b) Indicate number of participants with missing data for each variable of interest	5, 24, 26
		(c) *Cohort study*—Summarise follow-up time (eg, average and total amount)	
Outcome Data	15*	*Cohort study*—Report numbers of outcome events or summary measures over time	
		Case-control study—Report numbers in each exposure category, or summary measures of exposure	
		Cross-sectional study—Report numbers of outcome events or summary measures	6, 24, 26

Section and Item	Item No.	Recommendation	Reported on Page No.
Main Results	16	(*a*) Give unadjusted estimates and, if applicable, confounder-adjusted estimates and their precision (eg, 95% confidence interval). Make clear which confounders were adjusted for and why they were included	6, 26
		(*b*) Report category boundaries when continuous variables were categorized	6
		(*c*) If relevant, consider translating estimates of relative risk into absolute risk for a meaningful time period	
Other Analyses	17	Report other analyses done—eg analyses of subgroups and interactions, and sensitivity analyses	7
**Discussion**			
Key Results	18	Summarise key results with reference to study objectives	9
Limitations	19	Discuss limitations of the study, taking into account sources of potential bias or imprecision. Discuss both direction and magnitude of any potential bias	11
Interpretation	20	Give a cautious overall interpretation of results considering objectives, limitations, multiplicity of analyses, results from similar studies, and other relevant evidence	9
Generalisability	21	Discuss the generalisability (external validity) of the study results	11
**Other Information**			
Funding	22	Give the source of funding and the role of the funders for the present study and, if applicable, for the original study on which the present article is based	17

*Give information separately for cases and controls in case-control studies and, if applicable, for exposed and unexposed groups in cohort and cross-sectional studies.

Once you have completed this checklist, please save a copy and upload it as part of your submission. DO NOT include this checklist as part of the main manuscript document. It must be uploaded as a separate file.
